# A Novel CNN-Based Framework for Alzheimer’s Disease Detection Using EEG Spectrogram Representations

**DOI:** 10.3390/jpm15010027

**Published:** 2025-01-14

**Authors:** Konstantinos Stefanou, Katerina D. Tzimourta, Christos Bellos, Georgios Stergios, Konstantinos Markoglou, Emmanouil Gionanidis, Markos G. Tsipouras, Nikolaos Giannakeas, Alexandros T. Tzallas, Andreas Miltiadous

**Affiliations:** 1 Department of Informatics and Telecommunications, University of Ioannina, Kostakioi, 47100 Arta, Greece; kstefan@gmail.com (K.S.); tzallas@uoi.gr (A.T.T.); 2 Department of Electrical and Computer Engineering, Faculty of Engineering, University of Western Macedonia, 50100 Kozani, Greece; ktzimourta@uowm.gr (K.D.T.);; 3 School of Science & Technology, Hellenic Open University, 26335 Patra, Greece

**Keywords:** Alzheimer’s disease, frontotemporal dementia, CNN, deep learning, EEG, spectogram, FFT

## Abstract

**Background:** Alzheimer’s disease (AD) is a progressive neurodegenerative disorder that poses critical challenges in global healthcare due to its increasing prevalence and severity. Diagnosing AD and other dementias, such as frontotemporal dementia (FTD), is slow and resource-intensive, underscoring the need for automated approaches. **Methods:** To address this gap, this study proposes a novel deep learning methodology for EEG classification of AD, FTD, and control (CN) signals. The approach incorporates advanced preprocessing techniques and CNN classification of FFT-based spectrograms and is evaluated using the leave-N-subjects-out validation, ensuring robust cross-subject generalizability. **Results:** The results indicate that the proposed methodology outperforms state-of-the-art machine learning and EEG-specific neural network models, achieving an accuracy of 79.45% for AD/CN classification and 80.69% for AD+FTD/CN classification. **Conclusions:** These results highlight the potential of EEG-based deep learning models for early dementia screening, enabling more efficient, scalable, and accessible diagnostic tools.

## 1. Introduction

Alzheimer’s disease (AD) is a debilitating neurodegenerative condition and the most common form of dementia, particularly among older adults [1]. Dementia, in general, refers to a group of symptoms affecting memory, cognitive abilities, and social functioning to the extent that they interfere with daily life [2]. AD is the leading cause of dementia, accounting for an estimated 60–70% of cases worldwide [3]. As the global population continues to age, the prevalence of AD and other dementias is rising at an alarming rate. In 2020, it was estimated that over 50 million people were living with dementia globally, a figure projected to increase to 75 million by 2030 and more than double to 152 million by 2050 [4].

AD is a major public health challenge, not only due to its high prevalence but also because of its devastating impact on patients and families. The disease manifests initially with subtle memory impairments, particularly in short-term recall, and progressively worsens, leading to severe cognitive decline, disorientation, language difficulties, mood disturbances, and ultimately, a complete loss of independence [5]. As the disease advances, it impairs bodily functions, ultimately resulting in death. Despite decades of research, there is currently no cure for AD, and available treatments are primarily aimed at alleviating symptoms, with limited effectiveness [5]. The urgent need for early diagnosis and intervention has driven extensive research into new diagnostic tools and therapeutic strategies, including automated methods for screening of patients [6].

Frontotemporal dementia (FTD) is another significant form of dementia, distinct from AD, primarily affecting the frontal and temporal lobes of the brain. Unlike AD, which typically manifests in older adults, FTD often presents at a younger age, with the onset commonly occurring between the ages of 45 and 65 [7]. FTD is characterized by progressive neuronal loss in the frontal and temporal regions, leading to a range of symptoms that differ from those seen in AD. Patients with FTD may experience marked changes in personality and behavior, such as apathy, impulsivity, and socially inappropriate actions, along with language difficulties, including problems with speech and comprehension [8,9]. However, differentiating between FTD and AD can be challenging, particularly in the early stages, as there can be overlapping symptoms between the two conditions [10]. As the disease progresses, these symptoms intensify, severely impacting social and occupational functioning. FTD is less common than AD, accounting for approximately 10–20% of all dementia cases, but it is a leading cause of early-onset dementia. The progression of FTD is typically rapid, and the prognosis is poor, with patients often surviving only 6 to 8 years after the onset of symptoms. Like AD, there is currently no cure for FTD, and treatment options are limited to managing symptoms and providing supportive care. The differentiation between FTD and other forms of dementia, such as AD, is crucial for accurate diagnosis and appropriate care, underscoring the importance of advanced diagnostic tools, including neuroimaging and machine learning (ML) approaches.

The diagnosis of AD and other types of dementia is an elimination procedure, where other reasons of cognitive decline are ruled out before the final diagnosis. It is typically guided by the criteria outlined in the Diagnostic and Statistical Manual of Mental Disorders (DSM-5) [11]. The diagnostic procedure involves several steps, such as clinical evaluation of the patient’s history, cognitive evaluation, neurological examination, and neurophysiological assessments. Additionally, clinicians may seek to rule out other potential causes of cognitive decline through blood tests and brain imaging. Also, while the DSM provides a standardized framework for the diagnosis, the confirmation of AD needs certain clinical criteria such as accumulation of neuritic plaques and neurofibrillary tangles containing hyperphosphorylated tau proteins need to be met (which usually are confirmed postmortem) [12]. This diagnostic workflow is not only costly but also time-consuming, thus leading to significant neurodegeneration before proper treatment is administered to the patient [13].

The delay in diagnosis is critical, as early detection enables timely intervention, symptom management, treatment planning, and potentially slows disease progression [14]. In recent years, the importance of early diagnosis has been recognized not only from a clinical perspective but also from a scientific perspective, and efforts have been made on employing advanced imaging techniques such as magnetic resonance imaging (MRI) and positron emission tomography (PET) to detect early AD markers. While these techniques have enhanced the ability to detect AD, they are still costly, not widely accessible, and slow. Thus, the need for other faster, more affordable, and cheaper alternative biomarkers is eminent.

Combining such biomarkers with artificial intelligence techniques could establish a reliable screening system to accelerate diagnosis and support neurologists.

Alterations in brain activity and disruptions in neural networks are significant indicators in neurodegenerative diseases like AD and FTD. Although several methods exist to measure brain activity, they vary widely in terms of spatiotemporal resolution and practicality. Techniques such as single-unit recordings offer high spatiotemporal precision but are limited by their invasive nature. On the other hand, non-invasive methods like functional MRI (fMRI) and electroencephalography (EEG) allow for the assessment of brain activity without the need for surgical intervention. Despite its high temporal resolution, EEG has historically been underutilized due to its low spatial resolution and susceptibility to noise. However, recent advancements in computational techniques, such as low-resolution electromagnetic tomography (LORETA), have improved the ability to estimate the location of brain activity sources, thereby enhancing spatial resolution. Additionally, techniques like independent component analysis (ICA) and artifact subspace reconstruction (ASR) have been developed to mitigate external interferences (e.g., eye movements) and correct signals, making EEG an increasingly promising tool for diagnosing neurodegenerative diseases.

EEG is a diagnostic tool that quantifies the activity of the brain by capturing the electrical alterations of the cerebral cortex. It captures the electrical postsynaptic potentials that are produced by the brain neurons by measuring the potential difference between a set of electrodes that can either be placed on the scalp or be intracranial. During the last decade, quantitative EEG has been examined thoroughly as a clinical tool for the detection, quantification, and assessment of brain conditions and diseases such as epilepsy [15], Parkinson’s disease [16], and hepatitic encephalopathy [17]. Besides these diseases, it has also been evaluated on neurodevelopmental disorders and emotional conditions such as dyslexia [18] and stress. Last but not least, there is a growing interest in the scientific community in deploying EEG analysis in neurodegenerative disorders such as AD, due to the consistent findings of abnormal frequency patterns in the brain’s electrical activity. Specifically, alterations in the beta and theta frequency bands have been observed in AD patients, with increased theta power and decreased beta power often linked to cognitive decline and disease progression. These changes in EEG frequency content offer valuable biomarkers for early detection and monitoring of AD, making EEG an increasingly attractive tool for non-invasive diagnosis and research. The combination of EEG and artificial intelligence for the automatic detection of AD (or other dementia) could accelerate the screening procedure and relieve the medical clinics from a lot of preliminary manual work.

The nature of the EEG signals is complex, non-stationary, and non-linear; thus, a lot of different feature extraction methods have been explored in order to analyze the different EEG-related problems. The most common way of analyzing EEG is by decomposing it into its frequency components, namely the alpha, beta, gamma, delta, and theta frequency bands. Each of these bands is defined by a different frequency range and represents (or is associated with) different cognitive and neurophysiological processes [15]. For instance, the alpha band (8–12 Hz) is related to relaxation and/or a closed-eyes awake state. The beta band (13–30 Hz) is associated with active thinking, motor control, and alertness, typically increasing during tasks requiring concentration or movement. The theta band (4–8 Hz) is connected to memory encoding, drowsiness, and early stages of sleep, playing a role in working memory and emotional processing. The delta band (0.5–4 Hz) is most prominent during deep sleep and is involved in restorative processes and brain recovery. Finally, the gamma band (30–100 Hz) is thought to play a key role in higher cognitive functions, such as perception, attention, and the integration of sensory information. Alterations in these frequency bands, such as increased theta and decreased beta power, are often indicative of neurodegenerative conditions like AD. To capture the frequency content of a signal, computational signal processing methodologies derived from the Fourier transform are employed, such as the Fast Fourier Transform (FFT) [19] or the Welch power spectral density (PSD) [20]. Some more advanced methods attempt to capture the frequency content of the signal as a function of time, employing time–frequency decompositions such as the discrete wavelet transform (DWT) [21,22] or the empirical mode decomposition (EMD) [23].

In cases where brain connectivity patterns are central to understanding the underlying pathology, such as in schizophrenia [24] or major depressive disorder (MDD) [25], methodologies that analyze interactions between different brain regions are crucial. In these conditions, disruptions in functional and structural connectivity have been linked to cognitive and emotional dysfunctions. Techniques such as coherence analysis and phase synchronization [24] are employed to examine the temporal coordination between EEG signals from various brain regions. Furthermore, approaches rooted in graph theory are often applied to model the brain as a network, allowing researchers to study properties like network efficiency, modularity, and hubness, which can reveal important insights into brain dysconnectivity. For example, in schizophrenia, altered connectivity patterns in the default mode network and fronto-parietal network have been associated with cognitive deficits and symptoms such as hallucinations and delusions [26]. Similarly, in MDD, disrupted connectivity between limbic and prefrontal regions is thought to contribute to mood regulation problems [25].

Timely diagnosis and screening for AD remain critical for early intervention and symptom management, particularly in light of the global aging population and the disease’s increasing prevalence. ML has emerged as a pivotal solution to this challenge, offering scalable and automated approaches to analyze complex biomedical data such as EEG signals. Numerous ML algorithms have been explored in the literature, ranging from more conventional methods such as support vector machines (SVMs) [27] to more advanced techniques like gradient boosting [13]. However, deep learning techniques, particularly CNNs, have gained substantial popularity due to their ability to automatically learn hierarchical features directly from raw data, bypassing the need for handcrafted feature engineering [4]. CNNs, in particular, excel in generalizability and adaptability, as they effectively model spatial and temporal patterns in data [28]. Studies leveraging CNNs for EEG-based AD diagnosis have demonstrated promising results, showing their capability to capture disease-specific neural patterns with high accuracy and robustness [29]. These advancements position CNNs as a cornerstone of modern diagnostic frameworks and underscore their potential for enhancing the early detection of AD, forming the foundation for this study’s novel approach.

Various automatic methodologies employing ML architectures have been proposed in recent years to address the challenge of AD detection. However, many of these approaches suffer from limited generalizability due to small sample sizes, lack of dataset diversity, or the use of unpublished datasets, making reproducibility difficult. Additionally, inappropriate validation methodologies remain a significant issue. For example, the use of k-fold validation on epoched and overlapping data [27,30,31,32] often results in biased testing, where segments from the same subject inadvertently appear in both the training and test sets, leading to overly optimistic performance metrics. These limitations highlight the importance of employing validation methods, such as LOSO validation, that are specifically tailored to EEG-based studies [33,34]. There is a clear need for diagnostic tools that not only leverage advanced deep learning techniques but also adhere to rigorous validation standards, ensuring reliable, reproducible, and clinically translatable results.

In this study, we propose a novel methodology for classifying EEG signals to distinguish AD and FTD patients from CN controls. Our approach leverages a CNN architecture designed to analyze EEG-based biomarkers. Specifically, we preprocess the EEG signals using ICA and ASR artifact rejection techniques, followed by feature extraction via the FFT. The extracted features are converted into spectrogram-like image representations, where each row corresponds to an EEG channel and each column to frequency components in the 4–40 Hz range. These representations are then fed into a CNN model, which automatically extracts relevant patterns and reduces the dimensionality of the input data. The network is trained and validated using the leave-N-subjects-out (LNSO) methodology to ensure robust evaluation and avoid data leakage. The proposed model is also compared against state-of-the-art ML methods to highlight its superior performance in AD classification and its potential to generalize to other dementia types. While this study focuses primarily on AD classification performance optimization, future work could adapt the methodology to further enhance its capability for FTD diagnosis.

## 2. Materials and Methods

**Figure 1:**
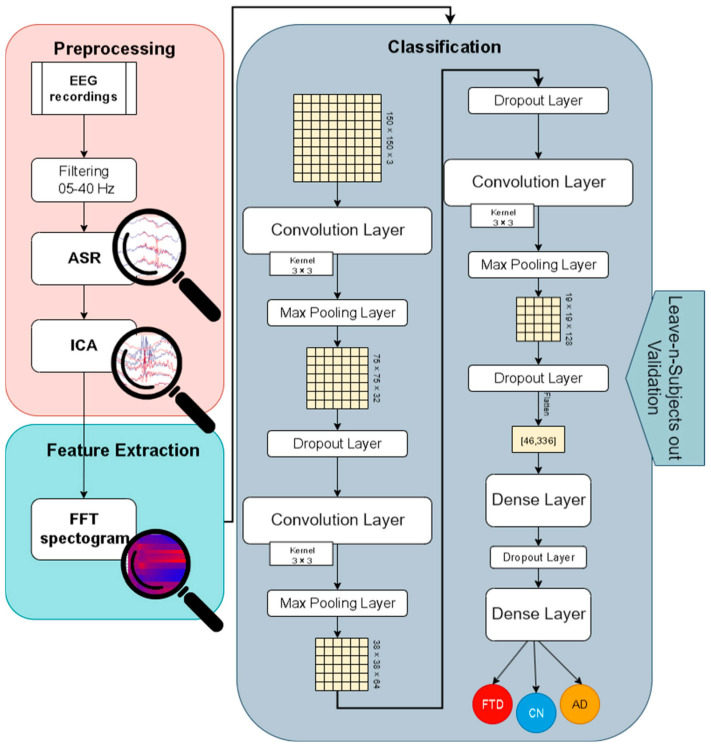
A detailed illustration of the methodology and system architecture.

In this paper, a robust methodology for the automatic discrimination of dementia-related EEG signals and healthy EEG signals is presented. This methodology is examined and compared along with other state-of-the-art EEG deep learning architectures, as well as other state-of-the-art ML algorithms, and is found to achieve superior results. In order to present the whole spectrum of the analysis, Section 2 is divided into four stages: Database Description, Signal Preprocessing, Feature Extraction, and Classification, which will be analyzed individually. Furthermore, the comparison experiments that took place will be presented in a brief manner. A detailed illustration of the methodology and system architecture is presented in Figure 1.

### 2.1. Database Description

In the following Section 2.1.1 and Section 2.1.2, a description of the demographic characteristics and the acquisition protocol of the EEG signals is provided. However, as we have also published these EEG recordings in compliance with the increasing demand for open data sharing and transparency, all of this information can be found in the accompanying data descriptor paper [35] we have published. This paper details the dataset, which is publicly available on the online data repository for BIDS-compliant data OpenNeuro [36], providing full documentation and metadata about the experimental setup, participants, and the signal acquisition process.

#### 2.1.1. Demographic Characteristics

The evaluation of the proposed methodology has been performed using EEG recordings from 88 participants acquired from the 2nd Department of Neurology of AHEPA General University Hospital of Thessaloniki. The approval of the Scientific and Ethics Committee of AHEPA University Hospital, Aristotle University of Thessaloniki was established under the protocol number 142/12-04-2023 and the whole procedure was carried out following the rules of the Declaration of Helsinki of 1975, revised in 2008. Informed consent for the use of their biomedical data was obtained from all participants.

Of the 88 participants, 29 were healthy (11 males and 18 females, denoted as the control group, CN), 36 were diagnosed with AD (13 males and 26 females), and 23 with FTD (14 males and 9 females). For every participant, the Mini-Mental State Examination (MMSE) [37] was calculated to evaluate their cognitive and neurophysiological state. The MMSE score ranges from 0–30, with higher scores indicating better brain functionality, and lower scores representing a more severe cognitive decline. A score of 30 represents a healthy individual. The AD group had an average MMSE score of 17.75 (±4.5), the FTD group had an average MMSE score of 22.17 (±8.22), while the CN group had perfect cognitive ability, thus a perfect MMSE score of 30.

Regarding the duration since the onset of the disease, it was reported in months with a median of 25 months and the interquartile range (q1–q3) being 24–28.5 months. Regarding the Clinical Dementia Rating (CDR) of the dementia groups, the average of the AD group was 1 (±0.54) and the average of the FTD group was 0.75 (±0.26). No dementia-related comorbidities have been reported for any participant. Finally, regarding the age of the participants, the mean age of the AD group was found to be 66.4 (±7.9), of the FTD group it was found to be 63.6 (±8.2), and of the CN group it was found to be 67.9 (±5.4). A summary of the demographic characteristics is presented inTable 1.

#### 2.1.2. Data Acquisition

EEG signals were recorded using a Nihon Kohden EEG 2100 clinical system, with 19 scalp electrodes positioned according to the international 10–20 system (Fp1, Fp2, F7, F3, Fz, F4, F8, T3, C3, Cz, C4, T4, T5, P3, Pz, P4, T6, O1, O2) and two reference electrodes (A1 and A2) placed on the ears. The recordings followed the clinical protocol in which participants were seated with their eyes closed. Prior and throughout the recording, skin impedance was kept below 5 kΩ. The sampling frequency was set at 500 Hz with a resolution of 10 μV/mm. Recording durations were approximately 13.5 min for the AD group (range: 5.1–21.3 min), 12 min for the FTD group (range: 7.9–16.9 min), and 13.8 min for the control group (range: 12.5–16.5 min).

### 2.2. Data Preprocessing and Signal Denoising

The EEG signals preprocessing actions were as follows. First, the signals were re-referenced to the A1–A2 electrodes. Next, Butterworth band pass filtering of 0.5–45 Hz was applied to ensure that only the frequency content of interest will be present and also that the power line noise interference at 50 Hz would be excluded.

Then, 2 automatic denoising methodologies were applied using the EEGLAB [38], Matlab U/I, namely the ASR [39] and the ICA [38]. ASR is an automatic artifact rejection methodology able to remove large-amplitude and transient artifacts [40]. Time periods of lousy data which exceeded the maximum acceptable standard deviation of 17 on a 0.5 s window were removed. The threshold of 17 was selected after examining the existing literature which suggests that a window of 10–30 is considered strict [41], and after manual experimentation with values over 10. Then, the electrode locations were established based on the channel names according to the “use MNI coordinate file for BEM dipfit model” option given in the EEGLAB tool (it should be noted that exact channel locations for each unique subject were not available, thus this was the best workaround). After channel locations were determined, the RunICA ICA algorithm was used to transform the 19 electrodes signal to 19 ICA components. These components were automatically labeled as brain components, eye artifacts, jaw artifacts, powerline artifacts, or other artifacts through the ICLabel pretrained routine that can be found in the EEGLAB. The components labeled as artifacts with a probability greater than 90% were excluded and the remaining components were used to recreate the cleared EEG signal.

**Figure 2:**
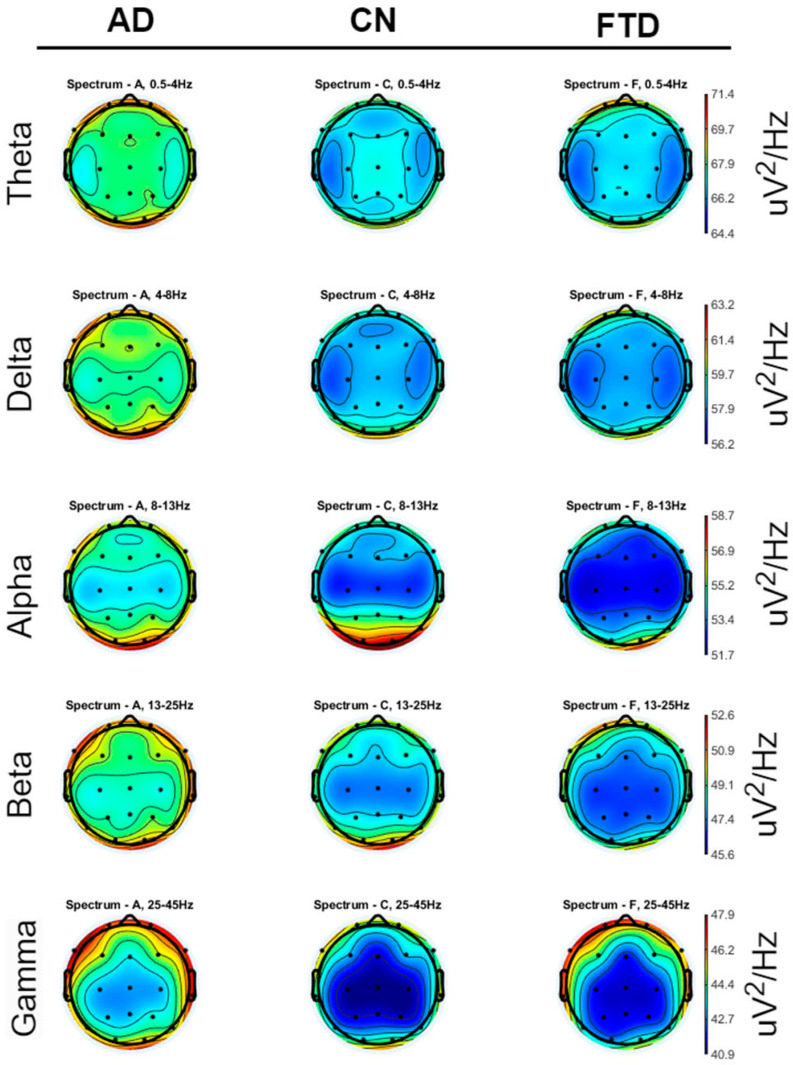
Scalp heatmaps of the average PSD of the 3 different classes, across the 5 frequency bands of interest.

To visually examine the data, the Welch power spectral density (PSD) was extracted for each subject by dividing it into the 5 frequency bands of interest, alpha (8–13 Hz), beta (13–25 Hz), gamma (25–45 Hz), delta (4–8 Hz), and theta (0.5–4 Hz), and averaged across all subjects of each class. Then, the average PSD of each group was expressed in a scalp heatmap configuration and is represented in Figure 2.

### 2.3. Feature Extraction

After the signal denoising step, having ensured that the data are as clear as possible, the data were epoched into 30 s epochs, in a total of 20 epochs per participant. Each epoched sample consisted of 15,000 time points, given the sampling rate of 500 Hz.

For each epoch, the FFT was applied for each of the 19 channels. The FFT algorithm computes the Discrete Fourier Transform more efficiently in terms of computational complexity (O(nlog⁡n) instead of $O\left(n^{2}\right)$). Given a series of *N* discrete time points, the DFT is defined as follows:

(1) $$X_{k}=\sum_{m=0}^{N-1}\;x_{m}e^{-i2\pi km/N}k=0,\ldots ,n-1$$

where $e^{i2\pi /N}$ is a primitive Nth root of 1.

To calculate the DFT the Cooley–Tukey FFT [42] algorithm was employed. The Cooley–Tukey algorithm works by recursively breaking down the DFT into smaller DFTs. For simplicity, let us consider the case where *N* is a power of 2, i.e., $N=2^{m}$. The algorithm splits the DFT into two parts: one for the even indices and one for the odd indices. Expressing the DFT recursively, the DFT can be split as follows:

(2) $$X_{k}=\sum_{m=0}^{N/2-1}\;x_{2m}\cdot e^{-i\frac{2\pi }{N/2}mk}+e^{-i\frac{2\pi }{N}k}\sum_{m=0}^{N/2-1}\;x_{2m+1}\cdot e^{-i\frac{2\pi }{N/2}mk}$$

and

(3) $$\begin{array}{c} E_{k}=\sum_{m=0}^{N/2-1}\;x_{2m}\cdot e^{-i\frac{2\pi }{N/2}mk}\;(DFT\;of\;even\text{-}indexed\;terms)\\ O_{k}=\sum_{m=0}^{N/2-1}\;x_{2m+1}\cdot e^{-i\frac{2\pi }{N/2}mk}\;(DFT\;of\;odd\text{-}indexed\;terms) \end{array}$$

We can express $X_{k}$ as $X_{k}=E_{k}+e^{-i\frac{2\pi }{N}k}\cdot O_{k}$, for k=0,1,…,N/2−1.

The recursive process continues until reaching the base case, typically a DFT of size 2, which can be computed directly.

**Figure 3:**
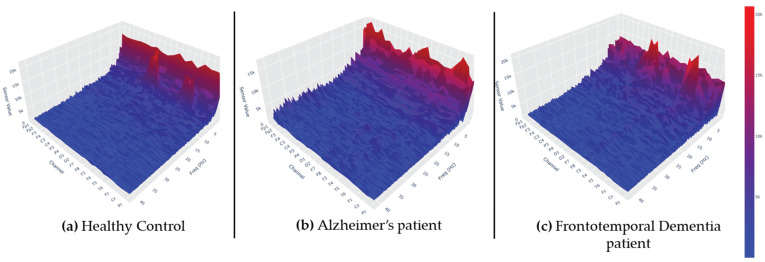
The charts depict the FFT transformation of one epoch for a participant from each of the three classes. The data are presented in a 3D representation to enhance visualization and provide a clearer view of the frequency distribution across channels (the original data were displayed as flat RGB images).

The FFT was calculated for each electrode, resulting in a 4D array of data for each epoch, with a frequency resolution of 0.033 Hz. Thus, these data were represented as RGB images, with color representing the frequency spectrum in μV. Figure 3 represents an epoch from each class in a more viewer-friendly appearance.

In total, 20 epochs were created for each participant, creating a data pool of 1760 images from the AD, FTD, and CN groups. The initial image size of each image was 19 × 1185.

### 2.4. Classification

In this section, the proposed deep learning architecture is described, the algorithms that were used to compare its performance are presented, and the validation method and performance metrics that were used are analyzed. It should be noted that the input shape of the images was transformed to rectangular 150 × 150 before feeding them to the classifier.

#### 2.4.1. Model Architecture

The dimensions of the input for the proposed CNN model are an RGB image of shape 150 × 150 (so, input shape 150 × 150 × 3). The proposed CNN model architecture begins with an initial convolutional layer utilizing 32 filters, each of size 3 × 3, with a linear activation function. To introduce non-linearity into the network, a LeakyReLU activation is applied, using an alpha value of 0.1 to allow a small gradient when the unit is not active. This is followed by a MaxPooling layer with a 2 × 2 pool size and the same padding to reduce the spatial dimensions, maintaining critical features while reducing computation. A Dropout rate of 0.25 is then applied to prevent overfitting by randomly setting a fraction of the input units to zero during training.

The model proceeds with a second convolutional layer, increasing the filter count to 64, while maintaining the same 3 × 3 kernel size and linear activation. Similar to the first layer, a LeakyReLU activation is used, followed by another MaxPooling layer with a 2 × 2 pool size and the same padding, and a Dropout rate of 0.25 to further reduce overfitting.

The third convolutional layer of the network increases the filter count to 128, utilizing the same configuration of 3 × 3 kernels and linear activation. The non-linearity is again handled by a LeakyReLU activation, followed by a MaxPooling layer with a 2 × 2 pool size and the same padding. To ensure robust feature extraction and avoid overfitting, a Dropout rate of 0.4 is employed at this stage.

Following the convolutional layers, the model includes a Flatten layer that transforms the multidimensional output from the previous layers into a one-dimensional vector, preparing it for the fully connected layers. A Dense layer with 128 units and a linear activation function is then added, followed by a LeakyReLU activation to introduce non-linearity. This dense layer is equipped with a Dropout rate of 0.3 to further guard against overfitting.

Finally, the network concludes with a Dense layer with 2 units, representing the output layer, which uses a softmax activation function. This activation function is suitable for multi-class classification as it converts the logits into probabilities, with each unit corresponding to one of the two target classes.

This final configuration of the CNN model was established after extensive experimentation with various architectures and hyperparameters in a series of ablation studies. Different configurations were tested by varying the number of layers, types of activation functions, Dropout rates, and other parameters to optimize the model’s performance. These systematic ablation experiments guided us to the current architecture, which achieved the best balance between accuracy, computational efficiency, and resistance to overfitting.

The number of epochs used for the training of the model was set to 100 (when referring to epochs in the context of neural network architectures, we mean the number of times the model obtained each instance of the training set as input, during the training step). This number was established after examining the train loss and the test loss, achieving low training loss but maintaining high generalizability.

#### 2.4.2. Comparison Algorithms

In order to evaluate the effectiveness of this methodology, other state-of-the-art deep learning architectures specifically designed for EEG classification, as well as other well-established ML algorithms, had to be examined for a comparison of their performance. In this section, these algorithms are enumerated, and later, in the Section 3, their performance is reported and compared with that of the proposed methodology. The EEG specific architectures tested were as follows:

1. EEGnet [43];
2. EEGNetSSVEP [44];
3. DeepConvNet [45];
4. ShallowConvNet [45].

Meanwhile, the non-deep learning, well-established ML methodologies examined were as follows:

1. XGBoost;
2. LightGBM;
3. Cat-Boost;
4. SupportVectorMachines on PCA-reduced dataset (PCA-SVM);
5. k-NN on PCA-reduced dataset (PCA-SVM);
6. MultilayerPerceptron (MLP), with 1 hidden layer of 96 neurons.

Every algorithm had its hyperparameters optimized through the Python library Hyperopt [46]. In order to train these algorithms, the same dataset was used but the epoching step was changed to 2 s epochs (in order to populate the dataset with a bigger number of samples). In order to have similar features but in a 2-dimensional (conventional table) format, the relative band power for each band of interest was extracted for each channel with the Welch PSD method.

#### 2.4.3. Validation Methodology, Classification Problems, and Performance Metrics

To validate the robustness of the proposed methodology, an LNSO validation methodology was employed. For each classification problem, each dataset group was split into 5 equal subsets. One subset from each group was left out to be the test set, while the remaining were used as the training set. This procedure was repeated recursively until all subsets were used as test set, and the total confusion matrix was created, from which the performance metrics were calculated.

In the case of epoched EEG datasets, the main issue with k-fold cross-validation compared to our validation strategy is the potential for data leakage. Since k-fold splits the data randomly across folds, data from the same subject may appear in both the training and validation sets. This can lead to an overestimation of performance, as the model may learn subject-specific features rather than generalizing to unseen subjects, which is the main goal in many EEG studies. In contrast, LNSO (and generally LOSO validation) ensures that each subject is fully separated between training and validation, providing a more accurate evaluation of cross-subject generalization.

The classification problems that were evaluated were as follows: AD/CN, FTD/CN, AD+FTD/CN, and AD/FTD/CN. For each binary classification problem, the reported metrics were as follows: accuracy (ACC), precision (PPV), recall (TPR), and F1 score (F1). For multiclass classification problems, PPV and TPR were not reported.

#### 2.4.4. Experimental Setup

The experimental protocol and the demographic characteristics can be found in the previous sections. Regarding the preprocessing and denoising of the EEG signals, it took place in the EEGLAB tool, while the FFT transformations and the creation of the heatmaps that were used as input in the CNN took place in TensorFlow-Python and MNE-Python, respectively. The Python version for all the procedures was 3.10 and the training of the models took place on a CUDA 11.7-supporting GPU, RTX 3060 Ti.

## 3. Results

In this section, the performance of the proposed model for each problem examined will be reported, along with the performance of the comparison algorithms.

**Figure 4:**
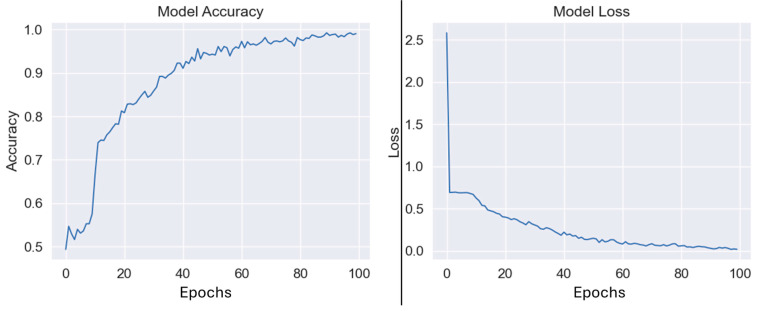
The train accuracy (**left**) and train loss (**right**) in the AD-CN problem, with respect to the number of epochs. An optimal performance is obtained at 100 epochs.

To evaluate the number of epochs that the neural network architecture should use, an 80–20% train–test split was established in the AD-CN and FD-CN problems and the epoch number where the test accuracy was maximized was selected. This number was found to be approximately 100 for both the problems evaluated, so it was selected as the epoch number for the training of the model for each LNSO run. Figure 4 presents the performance of the classifier with respect to the number of epochs for the AD-CN problem.

Table 2 presents the ACC results for each of the five batches for AD/CN, FTD/CN, and AD+FTD/CN, along with the average ACC across batches. Also, a 95% confidence interval is reported, along with the lower and upper bound.

As observed in the previous table, the reported ACC of the AD/CN problem was 79.45%, the reported ACC of the FTD/CN problem was lower, at 72.85%, and finally, the reported ACC for the combined AD+FTD/CN problem was the highest reported (also with the lowest standard deviation), at 80.69%. Regarding the standard deviation, the AD/CN had the highest reported at 7.06% while the other two problems achieved relatively low deviation at 3.08% and 2.25%, respectively, allowing an estimation of the 95% confidence interval with upper and lower bounds at 70.68–88.22% for the AD/CN problem, 69.02–76.67% for the FTD/CN problem, and 77.91–83.48% for the AD+FTD/CN problem.

The precision, recall, and F1 score performance metrics for each class, for these problems, are reported in Table 3.

As reported in the table, the best performance(s) in terms of precision, recall, and F1 score is/are achieved in the AD/CN and AD+FTD/CN problem, ranging from 74.8% to 76.15%, while the FTD/CN problem did not perform as well, with precision, recall, and F1 score ranging from 67.85% to 71.33% (approximately 5% lower). This may be attributed to either the fact that our methodology may not perform as effectively for distinguishing FTD cases, or the possibility that the FTD data were less distinguishable, potentially due to higher MMSE scores in the FTD group or higher variability within the group itself.

Regarding the multiclass AD/FTD/CN problem, although not the main goal of this study, the achieved performance is reported in Table 4. The reported accuracy was 54.28%, which is not great for a three-class problem. Again, taking into account the individual performances of each class in terms of precision, recall, and F1 score, it is apparent that FTD is the problematic class, with 33.74% individual precision, 19.55% individual recall, and 23.54% individual F1 score, making apparent that further exploration or better data are needed to make assumptions about the competence of this methodology on FTD detection, as it is further analyzed in the Section 4.

**Figure 5:**
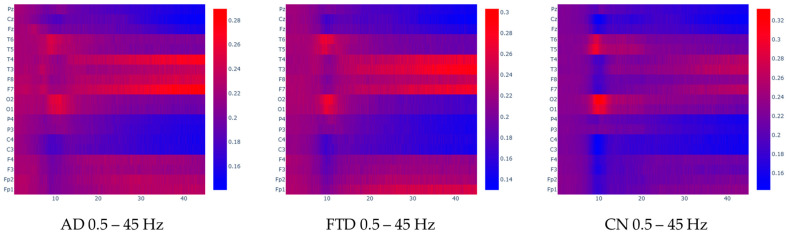
Averaged FFT heatmaps for each group in the 0.5–45 Hz frequency range. Each row represents a channel and each column represents a frequency value. The units are uV^2^/Hz.

**Figure 6:**
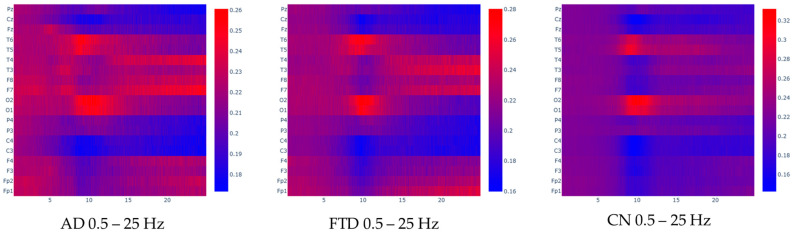
Averaged FFT heatmaps for each group in the 0.5–25 Hz frequency range. Each row represents a channel and each column represents a frequency value. The units are uV^2^/Hz.

To facilitate the visual inspection of the input data, the spectrogram heatmaps that were used as input for the model were separated in their respective classes and averaged, creating an average heatmap for each class. For better understanding, the following Figure 5 and Figure 6 represent the average frequency content of the classes in the 0.4–45 Hz range, but also in the 0.4–25 Hz range (which is expected to contain the most information related to the given problem, since the literature claims that AD is mainly associated with alterations in alpha, rhythm, or alpha–theta [47,48,49] and beta–theta ratios [10]. By visualizing these averaged spectrograms, we aim to highlight the characteristic differences in frequency content between the classes, which could provide insights into the distinct neural patterns underlying each condition. Observations regarding this averaged image analysis are provided in the Section 4.

To compare and validate the performance of the methodology, some well-established ML methodologies were examined using the same features, in 2 s epochs and with LOSO validation. Also, some state-of-the-art EEG-specific neural networks were examined using raw EEG signal as input, again with LOSO validation. All these algorithms and models have been reported in the Section 2. Their performance results of the ML algorithms that use the same extracted features are reported in Table 5 (AD/CN) and Table 6 (FTD/CN), while the performance of the raw-signal EEG neural networks are reported in Table 7 (AD/CN) and Table 8 (FTD/CN). The evaluation process for each algorithm was repeated 10 times and the averaged results are reported in the tables. The standard deviation of the proposed methodology results is presented in parentheses, and the * symbol next to the performance of a comparison algorithm indicates that the value was significantly lower than the corresponding proposed methodology value.

As observed, the proposed methodology outperforms all the other ML methodologies that were examined. In the AD/CN problem, the LightGBM achieved the best performance in terms of accuracy (76.28%) and F1 score (75.83%) yet being outperformed by the proposed methodology by 3.17% and 1.77%, respectively. The proposed methodology also outperformed all the comparison algorithms in terms of precision and recall.

Regarding the FTD/CN problem, the performance of the comparison algorithms was also lower than their respective performance on the AD/CN problem, indicating that the poorest performance on the FTD/CN problem is not model-related but rather data-related. The SVM-PCA algorithm achieved the best performance in terms of accuracy (70.93%) yet being outperformed by the proposed methodology by 1.92%. The MLP algorithm achieved the best performance in terms of F1 score (59.24%) yet being outperformed by the proposed methodology by 8.58%. The proposed methodology also outperformed all the comparison algorithms in terms of precision and recall, except the SVM-PCA, which achieved a precision of 75.26% while the proposed methodology fell short by 3.93%.

Regarding the performance of the state-of-the-art EEG neural networks, it is apparent that they failed in classifying the AD/CN and the FTD/CN problem. The highest accuracy on the AD/CN problem was 54.21% by DeepConvNet and the highest accuracy on the FTD/CN problem was 64.21%, again by the DeepConvNet, falling short by a staggering 25.24% and 8.64%, respectively. They are not well suited for the specific classification tasks and are outperformed by even the most basic ML methodologies on spatial features; thus, they should not be taken into consideration for the validation of the performance of our proposed methodology.

## 4. Discussion

A novel deep learning architecture for the automatic detection of AD and/or FTD signals was proposed in this study. The methodology consisted of the following: (a) a preprocessing step that employed basic preprocessing (filtering, re-referencing, etc.) and more computationally expensive artifact rejection methods (ICA and ASR), (b) a feature extraction step, which consisted of 30 s epoching, FFT calculation, and heatmap creation (where each row represented a channel and each column a frequency in the range of 4–40 Hz), which were used as input in (c) a deep learning convolution model which was implemented in TensorFlow and evaluated using the LNSO validation methodology. The performance of the model was compared with the performance of state-of-the-art raw-signal deep learning architectures specifically designed for EEG classification, as well as well-established ML algorithms with the same feature-extraction process, and was found to outperform them all in terms of accuracy and F1 score.

The findings of this study demonstrate the efficacy of the proposed methodology for detecting AD and/or FTD signals from EEG data. The model achieved superior performance compared to both state-of-the-art raw-signal deep learning architectures and well-established ML algorithms. Specifically, for the AD/CN and AD+FTD/CN classification tasks, the proposed architecture consistently outperformed all benchmarks in terms of accuracy and F1 score, with results ranging from 79.45% to 80.69%, depending on the task. These results highlight the robustness of the proposed methodology in identifying distinct neural patterns associated with AD and its ability to generalize across multiple classification problems.

In contrast, the performance for the FTD/CN problem was notably lower, with metrics such as accuracy (72.85%), precision (71.33%), recall (67.94%), and F1 score (67.85%), approximately 7% lower than for the AD/CN problem. This disparity may stem from two factors: (a) the inherently less distinguishable EEG features of FTD compared to CN, possibly due to overlapping spectral patterns or greater variability within the FTD group, and (b) the higher average Mini-Mental State Examination (MMSE) scores in the FTD group, suggesting less severe cognitive decline and subtler EEG alterations compared to AD patients.

For the AD/FTD/CN multiclass problem, the overall performance metrics were lower than the binary classification tasks. The average accuracy was 54.28%, with individual class metrics showing that FTD had particularly low precision (33.74%), recall (19.55%), and F1 score (23.54%). This is likely due to the challenges introduced by the FTD group, as their features seem to overlap significantly with both the AD and CN groups, leading to increased misclassifications. The results suggest that the EEG patterns of AD and CN are more distinct than those of FTD and either AD or CN, underscoring the need for more tailored methodologies or additional features to improve the classification of FTD cases.

These findings not only validate the proposed model’s capability for AD detection but also highlight the challenges faced in FTD-related tasks. Importantly, the observed limitations in the FTD/CN and AD/FTD/CN classifications are likely data-related rather than inherent to the model. This conclusion is supported by the fact that comparison methodologies, including both ML and deep learning approaches, exhibited similarly reduced performance for FTD-related tasks. If the issue were model-related, we would expect other methodologies to outperform the proposed model, which is not the case. Instead, the consistent drop in performance across all approaches suggests that the FTD data themselves may be less distinguishable, likely due to higher variability within the group, overlapping EEG patterns with other classes, or the relatively higher MMSE scores of FTD participants. Addressing these challenges will require better-quality or more extensive datasets, improved class balance, or the integration of additional biomarkers to enhance the differentiation of FTD cases. Nonetheless, the model’s strong performance in the AD/CN and AD+FTD/CN tasks establishes it as a robust tool for early dementia detection, with the potential to achieve even greater success with enriched data resources.

Regarding the visual inspection of the averaged spectrograms, distinct spectral patterns have been observed across the three groups. These patterns are in line with known neurophysiological changes associated with each condition.

In CN spectrograms, the alpha (8–12 Hz) and beta (13–30 Hz) bands display greater spectrum power, particularly in the posterior regions (occipital lobe: O1, O2; parietal lobe: P3; and temporal regions: T6, T5, T3, T4). This distribution is symmetrical and typical of a closed-eyes, calm condition. These participants’ states of alertness and restfulness are consistent with the alpha rhythm’s primacy in the occipital brain and its symmetrical propagation to the temporal regions. These results are in line with the typical EEG patterns seen in clinical recordings of healthy people.

The spectrograms indicate a generalized slowing of activity for AD patients, with increased theta activity (4–7 Hz) and decreased power in the alpha band (8–12 Hz). These changes are particularly evident in the posterior regions (O1, O2) and the temporal lobes (T6, T5), compared to healthy controls. Additionally, there is a notable asymmetry, with increased beta power in the right temporal lobe (T4) compared to the left, and increased power in the left frontal lobe (F7) compared to the right. This asymmetry reflects the often-uneven neurodegenerative progression in AD, particularly in frontotemporal regions. These spectral shifts highlight the disruption of normal neural oscillations and connectivity characteristic of AD pathology.

As for the FTD group, the spectrograms reveal a shift toward higher theta activity that is more diffused over frontal and temporal regions (T3, T4, F7, F8), along with a noticeable decrease in alpha power in the temporal and occipital regions, which is comparable to AD. Unlike AD, FTD displays slightly elevated power in the temporal and frontal regions, with reduced activity in posterior regions. The underlying neurodegenerative processes of FTD, which primarily impact the frontal and temporal lobes, are consistent with this pattern. Different pathogenic pathways are highlighted by the proportionate increase in power in the frontal and temporal regions when compared to AD, which helps distinguish the two disorders. The different neuronal oscillatory signatures linked to the HC, AD, and FTD groups are highlighted by these visual comparisons of averaged spectrograms. The observed patterns align with known pathophysiological changes.

We now further discuss the spectral patterns observed in Figure 5 and Figure 6 which provide valuable insights into the neurophysiological differences between the CN and the dementia groups (AD and FTD). These patterns reflect the disrupted brain dynamics associated with neurodegeneration, aligning with findings in the existing literature. Specifically, the CN demonstrates well-defined alpha activity localized to the occipital (O1–O2) and posterior temporal (T5–T6) regions, consistent with normal brain function during resting-state eyes-closed conditions. This localized alpha activity is a hallmark of healthy cortical networks, particularly in the posterior regions, which are involved in visual processing and memory functions [50]. The distinct drop in alpha power in other channels (e.g., frontal and central regions) further emphasizes the functional segregation in healthy brains [51]. In contrast, the dementia groups (AD and FTD) exhibit diffuse alpha activity across broader regions, including frontal, temporal, and central areas (e.g., T4–T3, F8–F7, P4–P3, F4–F3, Fp1–Fp2). This shift from posterior-dominant alpha activity to more widespread distributions aligns with the disrupted connectivity and desynchronization of neural networks often observed in neurodegeneration [4,52]. For AD, the shift of alpha power from posterior to anterior regions is a recognized phenomenon, reflecting the progressive atrophy of parietal and occipital areas and the involvement of more frontal regions as the disease advances [53]. The dementia groups also show an increase in delta and theta activity, which is consistent with established findings linking these slower oscillations to cortical dysfunction. Delta and theta power increases are strongly correlated with the degree of neurodegeneration and cognitive decline, particularly in regions affected early in the disease, such as the hippocampus and frontal lobes [10]. These findings are significant, as they highlight the potential of these spectral features as biomarkers for detecting and monitoring neurodegenerative diseases. Interestingly, the dementia groups also exhibit higher beta activity in regions such as T4–T3 and F8–F7. While this is less commonly reported, it could indicate compensatory mechanisms or increased neural noise resulting from disrupted cortical networks. Elevated beta power has occasionally been linked to maladaptive plasticity or hyperexcitability in response to neurodegeneration. This finding warrants further investigation, as it may represent an additional feature for characterizing these conditions or understanding their progression.

However, comparing the spectral patterns between AD and FTD proves more difficult. The heatmaps appear very similar, which aligns with the known challenge of differentiating these conditions based on EEG alone. Both disorders involve diffuse cortical dysfunction, leading to overlapping spectral changes [27]. However, subtle differences may exist in the frontal and anterior temporal regions (e.g., F7–F8, T3–T4), which are more prominently affected in FTD. FTD is characterized by localized degeneration in the frontal and anterior temporal lobes, whereas AD typically involves more posterior regions in earlier stages. While these differences may not be clearly discernible in Figure 5 and Figure 6, further analysis using advanced techniques or additional frequency bands may uncover more distinct features. For example, connectivity measures or non-linear analysis methods could provide deeper insights into the underlying pathophysiological differences.

To evaluate and benchmark our study, a comprehensive analysis of recent advancements in ML and DL applications for EEG-based dementia detection is required. A systematic review of neuroimaging approaches, including EEG, for AD detection highlights the increasing use of deep learning models such as CNNs and recurrent neural networks (RNNs). While these methods are promising, there are still challenges such as limited datasets and the need for effective feature representations. Unlike many prior studies that rely on raw EEG signals [54], our approach incorporates advanced preprocessing steps—specifically, ICA and ASR—to mitigate artifacts and enhance signal quality, and employs FFT-based feature extraction, offering a structured representation of frequency-domain information that is particularly relevant for distinguishing neurodegenerative conditions like AD and FTD.

Another notable trend in recent literature is the application of transfer learning for AD detection using EEG data [55,56]. Even though pretrained models are a viable solution in addressing the limitations of small datasets, as transfer learning allows for leveraging knowledge from related tasks, such methods are usually constrained by the variability in EEG data and the reliance on datasets that are not related to dementia-related signals. Our methodology refrains from this and relies solely on dementia-related EEG data which are problem-specific and publicly available for further exploration or validation. Last but not least, another trend in automated dementia detection is the combination of multiple biomarkers or modalities such as EEG, audio data, MRI, and others in complex, multimodal approaches [57,58]. These studies do achieve superior performance (for example, Sun et al. [58] combined EEG, MRI, and β-protein indicators and achieved a remarkable precision of 91.6% in estimating the Aβ1-42/Aβ-40 content through ML), yet they face issues regarding data integration, increased computational complexity, data availability, and acquisition speed, making them less practical for widespread application. Our approach, focusing solely on EEG data, maintains relatively high classification performance while ensuring minimum acquisition time, making it promising as a screening step in the AD detection protocol, particularly in resource-limited settings.

The novelty of our approach lies in combining advanced preprocessing, FFT-based feature extraction, and a specialized deep learning (DL) model. The use of ICA and ASR significantly enhances signal clarity, while FFT-based features effectively capture relevant spectral information linked to AD (and FTD). Our tailored CNN architecture leverages these features to achieve robust performance, outperforming both traditional ML models and state-of-the-art EEG-specific DL architectures in binary and multiclass classification tasks. The consistent drop in performance observed across all methodologies for FTD-related tasks further supports the argument that these challenges are data-related rather than model-specific. This highlights the importance of addressing dataset limitations to improve the detection of FTD cases. Our study not only aligns with the current advancements in EEG-based dementia detection but also addresses existing limitations through a comprehensive preprocessing pipeline and a specialized DL architecture. This results in a more accurate and reliable classification model, setting a new benchmark for EEG-based AD and FTD detection.

To provide a comprehensive understanding of our methodology’s effectiveness, it is crucial to compare it with other studies that utilize the same dataset in terms of methodology and performance. A detailed comparison not only highlights the strengths and potential of our approach but also identifies areas for improvement relative to existing techniques. In Table 9, we present a summary of related studies, showcasing their methodologies, reported performance metrics, and limitations. These include a variety of approaches such as entropy-based measures [31], dual-branch networks [30], and connectivity-based features [59], evaluated through differing validation methods. For instance, Velichko et al. achieved 88.45% accuracy using a novel entropy measure and SVM [31], while Rostamikia et al. reported a notable 93.5% accuracy for AD+FTD/CN classification using traditional time–frequency features and SVM [27]. Additionally, studies like Wang et al. leveraged aperiodic EEG components for AD/FTD classification with an AUC of 0.73 but relied on k-fold validation [32], which introduces potential limitations. Through this analysis, we aim to position our methodology within the broader research landscape, highlighting its performance and areas for future exploration.

A critical aspect in evaluating methodologies within the field of EEG-based dementia classification is the choice of validation strategy. As observed in Table 9, the majority of studies, including those employing advanced methodologies such as NNetEn [31] or dual-branch networks [30], rely on k-fold cross-validation for performance evaluation. While k-fold validation is a widely used approach, its application to epoched datasets can inadvertently lead to overly optimistic performance estimates. This occurs because epoched datasets often contain temporally overlapping or correlated segments from the same subject, which can inadvertently appear in both training and testing sets, violating the independence assumption of cross-validation.

In contrast, only two studies [59,60] adopted the more stringent LOSO, which ensures that data from each subject are exclusively used for either training or testing, thus providing a more realistic estimate of generalization performance. The consistent use of LOSO validation in future research is critical for reducing the risk of inflated metrics and ensuring robust and clinically translatable results. Greater awareness and caution in selecting validation methods will help address this methodological pitfall and enhance the reliability of findings across studies.

Having established the strengths and the effectiveness of our methodology, what is left is for the limitations to be addressed. The first and most important limitation regarding this study is the poor performance achieved in the FTD classification, in comparison to the AD classification. Although our methodology outperformed all the comparison algorithms in the FTD/CN problem (ACC = 72.85%, F1 score = 67.85%), these scores cannot be deemed effective and further research should be performed in order for them to be increased. Since the FTD/CN performance is poor across all algorithms, the issue appears data-related rather than model-related, underscoring the need to enrich the dataset with more diverse and higher-quality FTD recordings. Furthermore, another great limitation of this study is that it is ignorant of precursor AD states, which would be of outmost importance in the early diagnosis and intervention of AD. This is due to the fact that the dataset does not incorporate earlier state data such as mild cognitive impairment (MCI) data.

We acknowledge this limitation and are actively working to expand the published dataset (or develop a second version) with an adequate number of MCI recordings from the same hospital. Regarding the applicability of other time–frequency-based transforms besides the implemented FFT spectrogram, we acknowledge that alternatives such as the wavelet transform do offer better time–frequency resolution and could be applicable in the context of this research, but given the fact that in eyes-closed resting-state recordings, time-domain inconsistencies are what we are looking for, we preferred the more computationally affordable FFT solution. Moving on to more technical limitations, the connection of functional connectivity disruptions with AD has already been established in the literature [62]. Thus, connectivity data such as EEG coherence could have been employed in the study to try to achieve a greater classification performance. This has not been the case though, in this study, since it has not been found to be particularly additive in the classification performance of this dataset, in a previous study. Alternatively, the reader can check the importance of the EEG coherence in the classification of AD in the same dataset on our study cited here [4]. Last but not least, not exploring the potential effectiveness of our methodology on other types of dementia such as Lewy body dementia can be considered a limitation.

The proposed spectrogram-based convolutional architecture demonstrates significant potential for accurate dementia EEG signal screening. Future work could explore several directions to further improve its performance and generalizability. A key priority is the expansion of the dataset, which would enhance the model’s robustness and applicability across diverse populations. Additionally, efforts should focus on testing the model’s adaptability to varied EEG electrode setups, facilitating broader adoption in clinical environments. The incorporation of graph theory approaches could also be investigated to leverage spatial information from EEG channels, potentially improving the model’s ability to capture complex interchannel relationships. Also, we could explore the use of alternative time–frequency transforms, such as wavelet transforms or empirical mode decomposition, to capture localized time–frequency patterns and further enhance the model’s ability to analyze non-stationary EEG signals. Finally, the methodology could be extended to include other neurodegenerative diseases, such as Lewy body dementia, building on its existing application to FTD as examined in this study. These advancements hold promise for refining DL techniques in EEG-based dementia detection, with significant implications for clinical practice and patient care.

## 5. Conclusions

In this study, we explored the potential of using an FFT spectrogram-based CNN DL architecture for the automated detection of AD in EEG recordings, also evaluating its effectiveness on FTD detection. We evaluated the performance of the methodology on our previously published clinical dataset recorded at AHEPA General Hospital of Thessaloniki, Greece, and published on the OpenNeuro repository. The methodology consisted of advanced preprocessing steps for automated artifact rejection (namely ICA and ASR), transformation to frequency-domain spectrogram representations and employment of a deep CNN architecture, and it achieved state-of-the-art classification performance (ACC = 79.45%, F1 score = 77.60%), outperforming several baseline models that were used for comparison. These findings add to the growing body of literature utilizing EEG for the automated detection of neurological disorders like dementia, with the potential to assist clinicians in timely patient screening and improved monitoring of AD.

## Figures and Tables

**Table 1:** Demographics of the participants.

	Gender	Age	MMSE	CDR	Disease Duration in Months
AD	13/23	66.4 (7.9)	17.75 (4.5)	1 (0.54)	25 (9.88)
FTD	14/9	63.6 (8.2)	22.17 (8.22)	0.75 (0.26)	23 (9.35)
CN	11/18	67.9 (5.4)	30		

**Table 2:** Performance in terms of accuracy for the classification problems AD/CN, FTD/CN, and AD+FTD/CN.

Test Accuracy	AD/CN	FTD/CN	AD+FTD/CN
Batch 1	85.96%	78.12%	83.17%
Batch 2	70.31%	71.18%	78.28%
Batch 3	86.08%	70.27%	83.00%
Batch 4	74.15%	72.00%	79.76%
Batch 5	80.73%	72.66%	79.27%
**Average**	**79.45%**	**72.85%**	**80.69%**
Std	7.06%	3.08%	2.25%
95% CI	8.77%	3.82%	2.79%
Lower bound	70.68%	69.02%	77.91%
Upper bound	88.22%	76.67%	83.48%

**Table 3:** Precision, recall, and F1 score for each class, for the problems AD/CN, FTD/CN, and AD+FTD/CN. The final row of each section represents the averaged metric for all the classes of the problem.

		AD/CN	FTD/CN	AD+FTD/CN
Precision	AD	78.25%	-	-
	CN	74.39%	69.42%	66.81%
	FTD	-	73.24%	-
	AD+FTD	-	-	84.31%
	Total	76.32%	71.33%	75.56%
Recall	AD	79.23%	-	-
	CN	72.90%	83.40%	68.00%
	FTD	-	52.48%	-
	AD+FTD	-	-	84.31%
	Total	76.06%	67.94%	76.15%
F1 score	AD	78.67%	-	-
	CN	75.52%	75.53%	66.46%
	FTD	-	60.17%	-
	AD+FTD	-	-	83.18%
	Total	77.60%	67.85%	74.82%

**Table 4:** Performance in terms of accuracy, precision, recall, and F1 score for the multiclass classification problem AD/FTD/CN.

Test Accuracy	AD/FTD/CN			
Batch 1	57.58%	Precision	AD	55.71%
Batch 2	50.56%		CN	61.78%
Batch 3	63.06%		FTD	33.74%
Batch 4	51.47%		Total	50.41%
Batch 5	48.73%	Recall	AD	59.65%
Average	54.28%		CN	74.57%
Std	5.92%		FTD	19.55%
95% CI	7.36%		Total	51.26%
Lower bound	46.92%	F1 score	AD	57.00%
Upper bound	61.63%		CN	66.98%
			FTD	23.54%
			Total	49.17%

**Table 5:** Performance comparison of the proposed methodology in the AD/CN problem with well-established classification methodologies using the same feature vector in 2 s epochs. Asterisks (*) indicate performances that are statistically significantly lower than those of the proposed methodology (*p* < 0.05)

AD/CN	Accuracy	Recall	Precision	F1
LightGBM	76.28% *	75.08%	76.67%	75.83% *
XGBoost	75.53% *	75.08%	76.55% *	75.29% *
CatBoost	75.39% *	74.50% *	76.68%	75.05% *
SVM+PCA	73.75% *	70.51% *	74.60% *	74.89% *
PCA-kNN	72.52% *	70.30% *	77.41%	73.69% *
MLP	73.69% *	72.98% *	77.80%	75.31% *
Proposed Methodology	79.45% (1.2)	76.06% (1.54)	77.32% (2.21)	77.60% (0.98)

**Table 6:** Performance comparison of the proposed methodology in the FTD/CN problem with well-established classification methodologies using the same feature vector in 2 s epochs. Asterisks (*) indicate performances that are statistically significantly lower than those of the proposed methodology (*p* < 0.05)

FTD/CN	Accuracy	Recall	Precision	F1
LightGBM	69.13% *	51.57% *	65.72% *	57.79% *
XGBoost	69.22% *	52.02% *	65.71% *	57.44% *
CatBoost	68.66% *	47.41% *	66.02% *	55.19% *
SVM+PCA	70.93%	45.85% *	75.26%	56.98% *
PCA-kNN	67.80% *	41.50% *	66.82% *	51.20% *
MLP	69.98% *	53.60% *	66.21% *	59.24% *
Proposed Methodology	72.85% (2.47)	67.94% (2.51)	71.33% (1.91)	67.85% (2.22)

**Table 7:** Performance comparison of the proposed methodology in the AD/CN problem with state-of-the-art EEG-specific neural network configurations that take as input raw EEG signal.

AD/CN				
Model	Accuracy	Recall	Precision	F1
EEGNet	41%	47.20%	37.89%	42.04%
EEGNetSSVEP	51.46%	56.78%	47.65%	51.82%
DeepConvNet	54.21%	45.43%	48.71%	47.01%
ShallowConvNet	42.18%	46.50%	49.74%	48.07%
Proposed Methodology	79.45%	76.06%	77.32%	77.60%

**Table 8:** Performance comparison of the proposed methodology in the FTD/CN problem with state-of-the-art EEG-specific neural network configurations that take as input raw EEG signal.

FTD/CN				
Model	Accuracy	Recall	Precision	F1
EEGNet	46%	42.20%	45.21%	43.65%
EEGNetSSVEP	61.46%	53.51%	51.40%	52.43%
DeepConvNet	64.21%	62.41%	58.14%	60.20%
ShallowConvNet	46.38%	42.58%	42.37%	42.47%
Proposed Methodology	72.85%	67.94%	71.33%	67.85%

**Table 9:** Comparison of the methodology of studies that use the same dataset as the dataset used in this study.

Study	Year	Methodology	Performance	Limitations
Velichko et al. [31]	2023	New entropy measure: NNetEn along with SVM	ACC = 88.45% in AD/CN	k-fold validation
Chen et al. [30]	2023	Dual-branch network combining vision transformers (ViTs) and CNN	ACC = 80.23% in AD/FTD/CN	k-fold validation
Rostamikia et al. [27]	2024	Time, frequency, and time–frequency features; SVM	ACC = 93.5% in AD+FTD/CN, ACC = 87.5% in AD/FTD	k-fold validation
Wan et al. [60]	2024	CEEMD-enhanced microstate sequence non-randomness index, LOSO validation	Prediction of MMSE: R^2^ = 0.940	Derived metrics require further validation
Wang et al. [32]	2023	Use of aperiodic components such as aperiodic exponent and offset	AUC = 0.73 AD/FTD	k-fold validation, assumes accurate separation of periodic–aperiodic components
Ma et al. [59]	2024	EEG connectivity measures, phase locking index, SVM classifier, LOSO validation	AD/CN = 76.9%, FTD/CN = 90.4%	No comparison with advanced DL models
Nedelijkovic et al. [61]	2023	Hjorth parameters (activity, mobility, complexity), skewness, and kurtosis; dimensionality reduction via info gain, random forest, XGBoost, LNSO	AD/CN ACC = 76.5%, F1 = 77.5%	No comparison with advanced DL models

## Data Availability

Datasets related to this article can be found at https://openneuro.org/datasets/ds004504, hosted at OpenNeuro [36].
